# The Establishment and Verification of a Nomogram Model for Predicting the Overall Survival of Medullary Thyroid Carcinoma: An Analysis Based on the SEER Database

**DOI:** 10.3390/curroncol31010006

**Published:** 2023-12-22

**Authors:** Wankun Wang, Xujin Wang, Gang Che, Jincheng Qiao, Zhendong Chen, Jian Liu

**Affiliations:** 1 Department of Surgical Oncology, The First Affiliated Hospital, Zhejiang University School of Medicine, Hangzhou 310000, China; wkwang@zju.edu.cn (W.W.);; 2 Department of Gastroenterology, The Second Affiliated Hospital, Zhejiang University School of Medicine, Hangzhou 310000, China

**Keywords:** medullary thyroid carcinoma (MTC), SEER, nomogram, overall survival (OS)

## Abstract

(1) Background: This study aimed to establish a nomogram model for predicting the overall survival (OS) of medullary thyroid carcinoma (MTC) patients based on the Surveillance, Epidemiology, and End Results (SEER) database. (2) Methods: Patients with MTC in the SEER database from 2004 to 2015 were included and divided into a modeling group and an internal validation group. We also selected MTC patients from our center from 2007 to 2019 to establish an external validation group. Univariate and multivariate Cox regression analyses were used to screen for significant independent variables and to establish a nomogram model. Kaplan–Meier (K-M) curves were plotted to evaluate the influence of the predictors. The C-indexes, areas under the curves (AUCs), and calibration curves were plotted to validate the predictive effect of the model. (3) Results: A total of 1981 MTC patients from the SEER database and 85 MTC patients from our center were included. The univariate and multivariate Cox regression analyses showed that age, tumor size, N stage, and M stage were significant factors, and a nomogram model was established. The C-index of the modeling group was 0.792, and the AUCs were 0.811, 0.825, and 0.824. The C-index of the internal validation group was 0.793, and the AUCs were 0.847, 0.846, and 0.796. The C-index of the external validation group was 0.871, and the AUCs were 0.911 and 0.827. The calibration curves indicated that the prediction ability was reliable. (4) Conclusions: A nomogram model based on age, tumor size, N stage, and M stage was able to predict the OS of MTC patients.

## 1. Introduction

In recent years, the prevalence of thyroid cancer has increased, year by year, making it the fastest-growing solid tumor in terms of prevalence [1,2]. Based on pathological type, thyroid cancer can be divided into papillary thyroid carcinoma (PTC), follicular thyroid carcinoma (FTC), medullary carcinoma, and undifferentiated carcinoma [3]. Among these types, the increase in the incidence of thyroid cancer is mainly attributed to the overdiagnosis of PTC in clinics, while the incidence of MTC is relatively stable [4]. MTC is a rare neuroendocrine malignant tumor that arises from parafollicular C-cells, accounting for 3% of all thyroid cancer cases [5]. It has been reported that approximately 15–20% of MTC patients exhibit distant metastatic disease at the time of diagnosis, and the 10-year overall survival rate after the first distant metastasis is approximately 10–40% [6].

Regarding MTC, 75% of instances are sporadic and the other 25% are hereditary [7], consisting of familial MTC and multiple endocrine neoplasia (MEN) types IIA and IIB [8]. It is generally believed that a specific autosomal dominant gain-of-function mutation in the RET proto-oncogene causes hereditary MTC [9]. Among the many mutations in RET codons, the most frequent is the mutation of the 918 codon [10]. MTC patients with RET 918 mutations are more likely to have extensive lymph node metastases in the neck and mediastinum as well as distant metastases in the liver, lungs, and bone [11].

The characteristics of MTC include slow growth and early susceptibility to lymph node metastasis, which are observed in approximately 35% of MTC patients at the time of initial diagnosis, affecting the neck and mediastinal lymph nodes [12]. Distant metastasis of MTC mainly occurs in the lungs, liver, and bones, and studies have confirmed that death related to MTC is mainly related to it. Nevertheless, studies have also shown that MTC has an average 15-year survival rate of around 85% [8]. Biochemical markers, clinical characteristics, and somatic mutation statuses can be used to predict prognosis and survival in MTC patients [13]. Calcitonin (CT) and the carcinoembryonic antigen (CEA) are important biochemical markers for the diagnosis and prognosis of MTC [14]. Recently, Nigam et al. established an international grading system for MTC based on the mitotic rate, Ki-67, and tumor necrosis. The grading system is divided into low and high grades, with the high grade generally predicting a more severe disease and a worse prognosis. In that study, it was observed that calcitonin levels doubled in most high-grade MTC patients within two years after surgery. Additionally, calcitonin/CEA doubling time and tumor grade can be used as independent predictors to assess the prognosis of MTC patients, thereby detecting disease recurrence [15].

The cure and survival rates of MTC patients are closely related to the stage at diagnosis [16]. The most commonly used staging system for MTC is the American Joint Committee on Cancer (AJCC) Tumor/Node/Metastasis (TNM) classification system, but this system lacks some factors that can help predict the prognosis of patients. Nomograms have been accepted as a reliable alternative tool that can help clinicians to make more precise predictions for individual patients [17].

Previous studies have confirmed the prognostic factors of MTC, including age, tumor size, N stage, M stage, calcitonin, and CEA level. However, the records of calcitonin and the CEA are incomplete in the SEER database. Furthermore, no nomogram model has been established to predict the prognosis of MTC patients [18]. Other studies have created nomogram models that have only contraposed patients who underwent surgery [19].

Therefore, we established a nomogram model to predict the OS of MTC patients based on clinical features and the TNM staging system. We constructed this model with data from the SEER database and took part of it for internal verification. The MTC patients from our medical center were analyzed retrospectively for external verification.

## 2. Materials and Methods

### 2.1. Data Selection from the SEER Database

We used SEER*Stat software (version 8.4.1.2; National Cancer Institute, Bethesda, Rockville, MD, USA), which was last updated in November 2022, and downloaded the data from the SEER database. The inclusion criteria were as follows: (1) patients diagnosed with MTC between 2004 and 2015 and (2) historical codes 8345/3 and 8510/3 on the basis of the International Classification of Disease for Oncology, third edition (ICD-O-3). We extracted the following clinical characteristics from the SEER database: age at diagnosis, sex, race (white, black, Asian or Pacific Islander, and American Indian/Alaska Native), year of diagnosis, tumor size, AJCC stage, TNM status derived from the AJCC system, survival months, and survival status. The exclusion criteria were the following: (1) patients with unknown or missing clinical information; (2) patients with survival times of less than one month; and (3) patients who died of other causes except MTC or had absent information on the cause of death. Finally, according to the inclusion and exclusion criteria, 1981 patients were selected from the SEER database.

### 2.2. Data Selection from Our Medical Center

We selected 85 patients with MTC from our medical center as the external validation group for this model. The pathological results were jointly diagnosed by two pathologists in our hospital. We recorded these patients’ ages at diagnosis, sexes, races (white, black, Asian or Pacific Islander, and American Indian/Alaska Native), years of diagnosis, tumor sizes, AJCC stages, TNM statuses derived from the AJCC system, survival months, and survival statuses. The exclusion criteria were as above.

### 2.3. Statistical Analyses

Microsoft Excel 2016 was used for data collection and recording. Statistical analysis was performed using R studio (Version 4.2.0). Kaplan–Meier (K-M) survival curves were compared using the log-rank test. *p*-Values of less than 0.05 were considered statistically significant. Univariate and multivariate Cox regression analyses were performed using R Studio, and the hazard ratio (HR) and 95% confidence interval (CI) were calculated. Both the HR and 95% CI reserved three decimal places. First of all, univariate Cox regression analysis was performed for each factor. Factors with *p* < 0.05 were incorporated into the multivariate Cox regression analysis. Through the above analysis, independent factors affecting the OS of MTC were screened out. We constructed a nomogram model with these independent factors. The data in the SEER database were randomly divided into a modeling group (*n* = 1389) and an internal validation group (*n* = 592) at a 7:3 ratio. In total, 85 MTC patients in our medical center formed an external validation group. We used Harrell’s concordance index (C-index), the receiver operating characteristic curves (ROCs), and the areas under the ROC curve (AUCs) to evaluate the exact prognostic performance of this nomogram. The prediction ability of a nomogram model is better when its C-index is closer to 1. Finally, the calibration curves were used to verify the consistency between the predicted OS and the actual OS.

## 3. Results

### 3.1. Clinical and Pathological Characteristics of Patients

According to the inclusion and exclusion criteria, a total of 1981 patients registered with MTC between 2004 and 2015 were selected from the SEER database for our study. The patients were divided into a modeling group (*n* = 1389) and an internal validation group (*n* = 592) at a 7:3 ratio. We reviewed the clinical characteristics of the 85 MTC patients from 2007 to 2019 at the Department of Surgical Oncology at the First Affiliated Hospital of Zhejiang University School of Medicine, which established an external validation group. The relevant clinical characteristics are recorded in Table 1.

### 3.2. Selection of Independent Factors for the OS and K-M Curves

**Figure 1:**
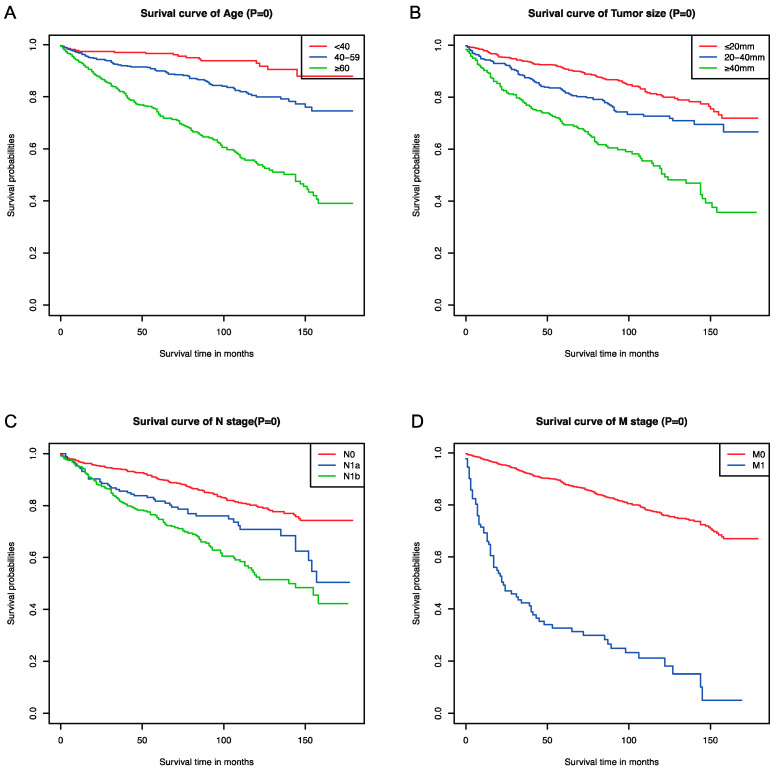
Kaplan–Meier survival curves of the significant independent factors: (**A**) age, (**B**) tumor size, (**C**) N stage, and (**D**) M stage.

Univariate and multivariate Cox regression analyses were used to select the factors that had significant influences on the OS. The hazard ratios (HRs) and 95% confidence intervals (CIs) retained three decimal places, and *p*-values of <0.05 were considered statistically significant. Finally, we found that age, tumor size, N stage, and M stage had significant impacts on the OS of MTC patients (Table 2). The K-M curves were drawn according to the selected independent factors (Figure 1).

### 3.3. Nomogram Development and Validation for the Prediction of the Overall Survival of MTC Patients

**Figure 2:**
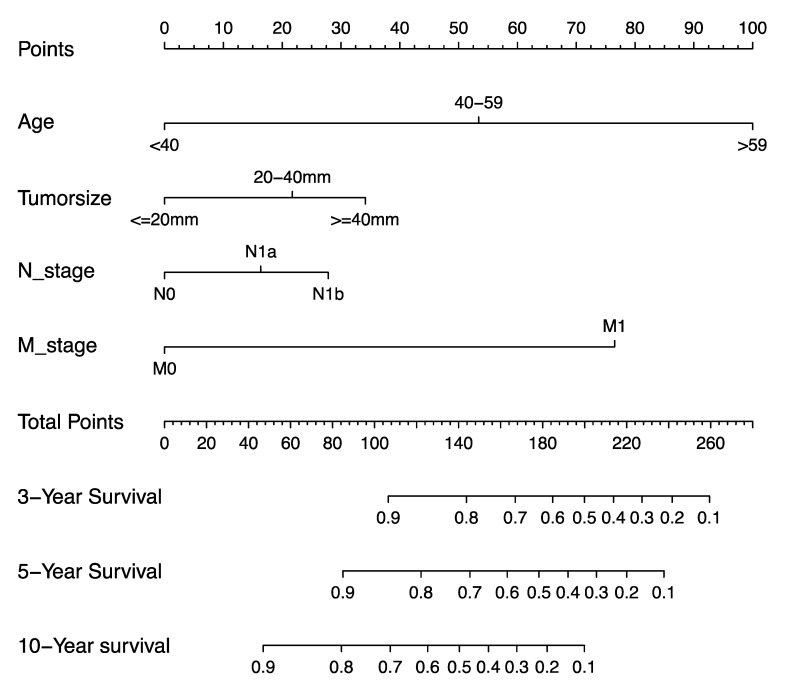
Nomogram for predicting the prognosis of MTC patients.

According to the results of the univariate and multivariate Cox regression analyses in the previous step, we extracted four factors to establish the nomogram model for predicting the OS of MTC patients, including age, tumor size, N stage, and M stage (Figure 2).

**Figure 3:**
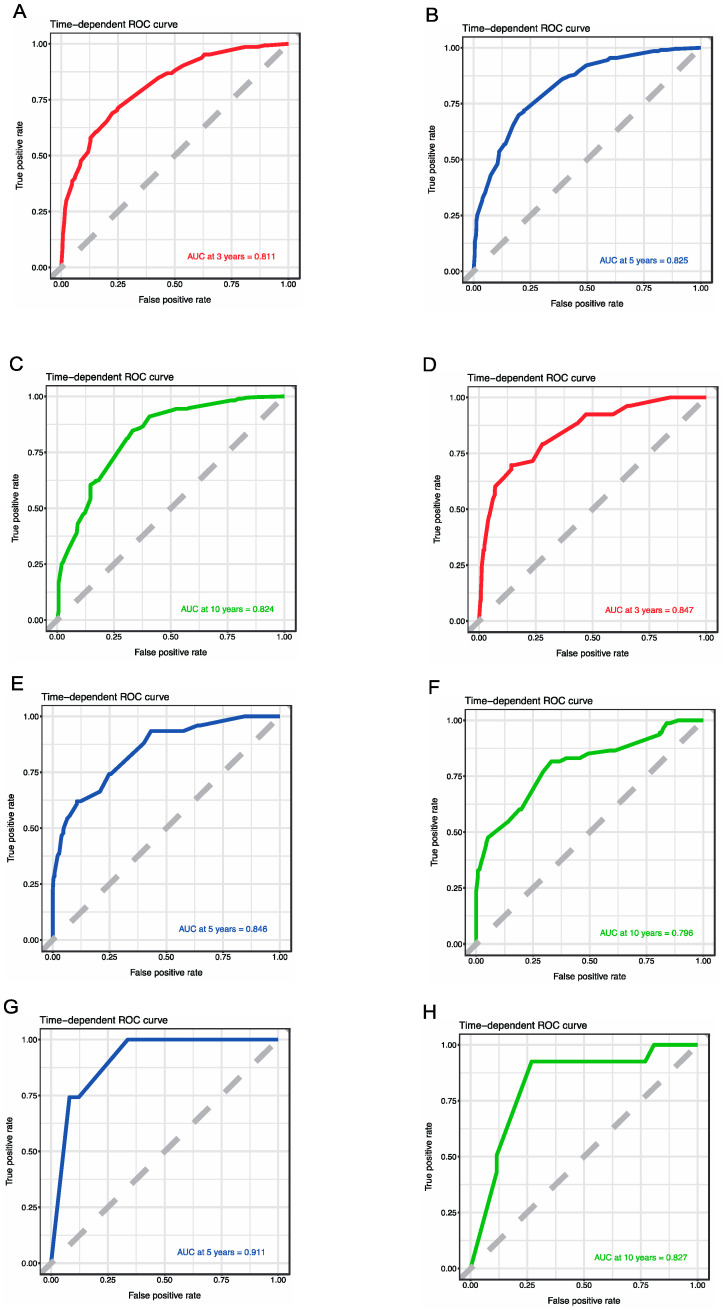
ROCs for assessing the accuracy of predicting the 3- (**A**), 5- (**B**), and 10-year (**C**) survival in the modeling group; ROCs for assessing the accuracy of predicting the 3- (**D**), 5- (**E**), and 10-year (**F**) survival in the internal validation group; and ROCs for assessing the accuracy of predicting the 5- (**G**) and 10-year (**H**) survival in the external validation group.

For the sake of correcting the bias of overfitting outcomes from testing on the same patient population, we selected data from the SEER database for internal validation. The prediction effect of a nomogram model can be demonstrated with the C-indexes, ROCs, and AUCs. The C-index of the modeling group was 0.792, and the AUCs of the 3-, 5-, and 10-year OS rates were 0.811, 0.825, and 0.824, respectively. The C-index of the internal validation group was 0.793, and the AUCs of the 3-, 5-, and 10-year OS rates were 0.847, 0.846, and 0.796, respectively. We selected 85 patients with MTC from our medical center as the external validation group for this model. The C-index of the external validation group was 0.871, and the AUCs of the 5- and 10-year OS were 0.911 and 0.827, respectively (Figure 3).

**Figure 4:**
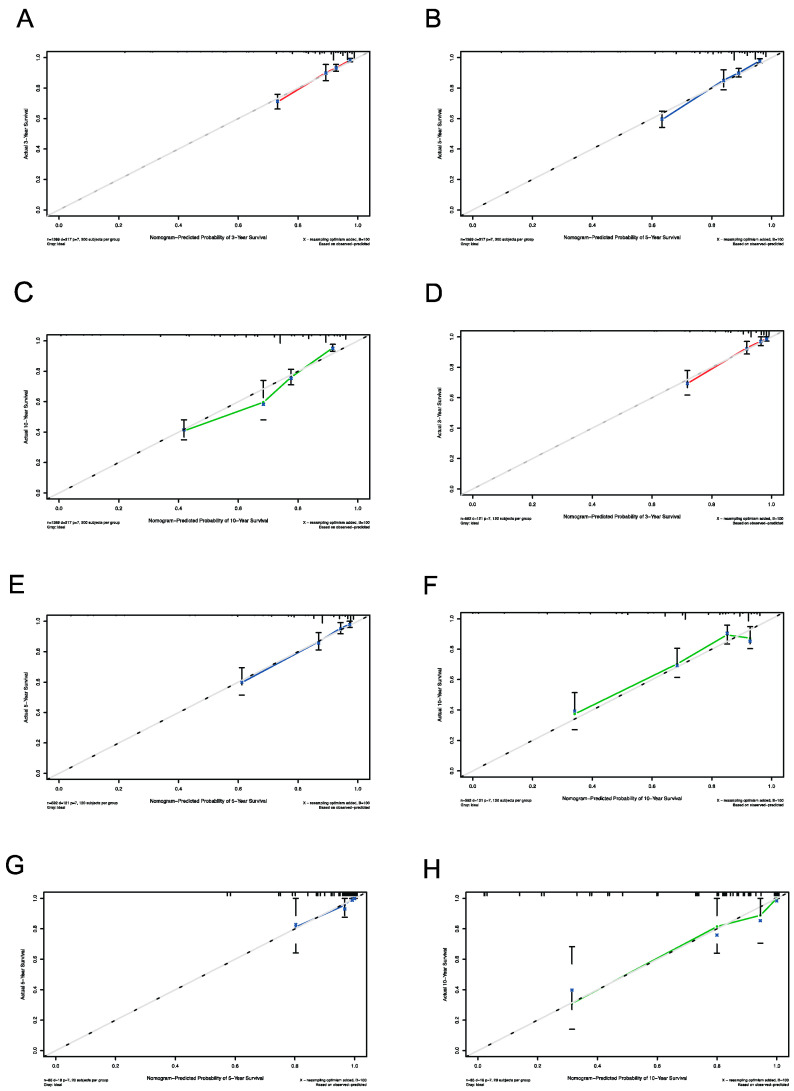
The calibration curves for predicting patient survival at 3 (**A**), 5 (**B**), and 10 years (**C**) for the modeling group. The calibration curves for predicting patient survival at 3 (**D**), 5 (**E**), and 10 years (**F**) for the internal validation group. The calibration curves for predicting patient survival at 5 (**G**) and 10 years (**H**) for the external validation group.

The calibration curves were used to verify the consistency between the predicted OS and the actual OS, indicating that the modeling group, internal validation group, and external validation group were in good agreement with the actual OS (Figure 4).

## 4. Discussion

MTC is a rare neuroendocrine malignant tumor that arises from parafollicular C-cells, accounting for 3% of all thyroid cancer cases [5]. Compared to PTC and FTC, MTC is a rarer pathological type and has a poorer prognosis [20]. It has been reported that approximately 15–20% of MTC patients develop distant metastasis, and the 10-year overall survival after the first distant metastasis is approximately 10–40% [16]. Regarding MTC, 25% of cases are hereditary MTC, divided into three subtypes: MEN IIA, MEN IIB, and FMTC [8]. MEN IIA is characterized by MTC, pheochromocytoma, and primary hyperparathyroidism. The clinical features of MEN IIB are mainly aggressive MTC, pheochromocytoma, and gangliocytoma. FMTC accounts for approximately 10–20% of hereditary MTC cases [9].

MEN II syndrome is an autosomal dominant inherited tumor syndrome with a prevalence of approximately 1/30,000 of the population [21]. In 1996, Eng et al. revealed the pathogenicity of RET proto-oncogene mutations in MEN II syndrome and found that codon 768 and 804 mutations only occurred in familial MTC (FMTC), while codon 918 mutations were specific to MEN IIB [22]. RET mutations have occurred in 70% of MTC patients [23]. Somatic RET mutations have been demonstrated to be associated with worse prognosis, and such patients are more likely to have lymph node metastasis at initial diagnosis and have lower survival rates [11]. Therefore, gene screening for RET has great significance for patients with family histories of MEN II syndrome and sporadic MTC.

Surgery has been the primary treatment for MTC. However, scientists have made new breakthroughs in treatment options for patients who cannot be operated on immediately. In recent years, some studies have found that targeted treatment of RET can inhibit the activation of tyrosine kinase receptors, thus inhibiting the growth of MTC cells [24]. Currently, the nonselective RET inhibition of multikinase inhibitors (MKIs) includes sorafenib, lenvatinib, vandetanib, cabozantinib, and sunitinib. However, due to the lack of the specific targeting of the RET gene, these drugs lead to a high incidence of drug-related toxicity, resulting in reduced efficacy or withdrawal [25]. Therefore, highly efficient, small-molecule, ATP-competitive, and highly selective RET inhibitors, including selpercatinib and pralsetinib, which aim to conquer gatekeeper RET mutations, have emerged in recent years [26]. In 2021, pralsetinib was approved by the FDA to treat lung and thyroid cancers with mutations or fusions of the RET gene [27]. In phase I–II clinical trials, selpercatinib showed a more durable effect and was less toxic [23]. Phase III clinical trials of selpercatinib for RET-mutated MTC are ongoing and may lead to a more personalized option for patients in clinical practice (NCT04211337) [28].

Recently, some scholars have proposed that dynamic risk stratification (DRS) has more predictive value than static anatomical staging systems like the AJCC TNM system. They added biochemical markers, such as calcitonin and the CEA, after initial treatment, as well as imaging tests, such as ultrasound, after treatment. Thus, more reliable information could be provided to evaluate MTC prognosis [29]. However, that study used single-center data and had a small sample size, which may have affected the generalizability of the results. In a retrospective study based on the SEER database, stage and age at diagnosis were strong predictors of survival in patients with MTC [18]. In our study, this was confirmed with data from the SEER database and from our medical center. We conducted modeling and internal validation with a large sample from the SEER database and external validation with local data. The C-indexes, AUCs, and calibration curves were used to prove that the nomogram model has good prediction ability. This model can be used at the first diagnosis of the disease, enabling clinicians to make more appropriate treatment plans for patients in practice.

In this study, we found that age, tumor size, N stage, and M stage were significant prognostic factors for MTC patients. First of all, we used univariate and multivariate Cox regression analysis to select the factors with significant influence. Since the MTC patients included in the external validation group from our center were all Asian, race was not included in the nomogram as an influencing factor. Some previous retrospective studies have suggested that male sex is one of the factors related to poor prognosis of MTC, but our study did not confirm gender as having a significant impact on OS [30]. Further verification via prospective research is required. According to the AJCC TNM staging system, all tumors with diameters between 2 and 4 cm are considered to be at the T2 stage. Our study confirmed that the prognosis of MTC in the T2 stage is indeed worse than that in the T1 stage. The AJCC TNM staging system has classified lymph nodes with unilateral or bilateral metastasis to area VI or VII (pretracheal, paratracheal, prelaryngeal/Delphian, or superior mediastinum) as N1a and classified lymph nodes with unilateral, bilateral, or contralateral cervical (I, II, III, IV, or area V) or retropharyngeal metastasis as N1b. Jiang et al. proved that a lymph node ratio (LNR) ≥76.5% is significantly associated with poorer OS [31], which is similar to what we found.

The common distant metastatic sites of MTC include the lungs, liver, and bones [32], which is in agreement with the data from our center’s follow-up. Among the 85 MTC patients followed up on in our center, eight cases had distant metastasis; this was a small sample and may have had a certain impact on the results.

However, there were still many limitations to this study. In terms of data collection, this research still has great opportunities for improvement. First of all, both the modeling group data and the internal validation group data were obtained from the SEER database. Although the SEER database collects data on cancer incidence, survival, and treatment across the United States and has a large sample size, it still lacks some laboratory data, such as calcitonin and the CEA, that are associated with MTC. The latest version of the SEER database has recorded the CEA data of some patients after 2010, but the CEA data of the MTC patients included in our study from 2004 to 2015 were all blank. Calcitonin is not recorded in the SEER database. This has resulted in incomplete data, preventing us from incorporating a large amount of biomarker data into our modeling and affecting the accuracy and completeness of our findings. Moreover, as this study was retrospective, it was not possible to develop a standardized procedure for the treatment of patients. In the study data from our medical center, biomarker measurements such as calcitonin and the CEA were not recorded regularly, so we could not obtain complete data to include in our study. This means that this study is potentially subject to more selection and information biases. Because this was a single-center study, publication bias due to small sample effects may have occurred. We can test publication bias by drawing funnel plots or via Egger’s test [33].

For the data used for external validation, only a small, single-center sample size was used. The retrospective analysis of the single-center data in this study may have general significance for the patients in our medical center, but it is still not applicable to a large number of patients. At the same time, the results of this study lack a certain generalizability.

In view of the above analysis, we also need to optimize and adjust these research methods. We need to conduct prospective multicenter studies with large sample sizes in the future. Multicenter studies require more capital investment, and a lack of financial support was also one of the limitations of this research. Multicenter studies can also provide a large amount of data but also bring many other challenges.

First of all, multicenter studies lack standardization of the study process. Due to the different inspection techniques and collection standards of each center, it is difficult to achieve the complete unification of data collection and analysis. This may affect the reliability and validity of findings. Multicenter studies also present some potential conflicts of interest. For example, different research centers will have different priorities, which also contributes to the progress and results of studies.

The nomogram is a statistical tool that can provide the most accurate predictions using a simple graphical presentation [34]. Nomogram models have been widely used in clinics and can solve the complexity of balancing different factors, allowing physicians to quantify a patient’s prognostic risk based on charts [35]. In 2020, Li et al. used age, metastasis, tumor size, and LNR to construct a nomogram model to predict the prognosis of patients after surgery [19]. However, only MTC patients who underwent total thyroidectomy and neck lymph nodes were selected for this study. It is difficult to use this nomogram model to predict the prognosis of some patients who are not eligible for surgery.

We constructed a nomogram model to predict the OS of MTC patients based on four factors: age, tumor size, N stage, and M stage. Internal and external validation were performed in the modeling and validation groups, respectively. The C-indexes, ROCs, and AUCs were used to evaluate the exact OS performance of the nomogram. The closer the C-index was to 1, the more accurate the predictive power of the model was. According to the follow-up data of our center, all patients survived for three years after the diagnosis of MTC, and there were no deaths, so the three-year ROC and its calibration curve were not drawn. The C-indexes of the modeling, internal validation, and external validation groups were 0.792, 0.793, and 0.871, respectively. Verification with the calibration curves showed that the OS predicted with the nomogram was in good agreement with the actual OS. This indicates that this nomogram model can accurately predict the OS of MTC patients and can help doctors provide more accurate treatment plans in future clinical practice.

## 5. Conclusions

We established a nomogram model to predict the OS of MTC patients based on four factors: age, tumor size, N stage, and M stage. This model can accurately predict the OS of MTC patients, thus enhancing patient counseling regarding clinical prognosis and follow-up.

## Figures and Tables

**Table 1:** Clinical and pathological characteristics of the MTC patients.

	Modeling Group (%) (*n* = 1389)	Internal Validation Group (%) (*n* = 592)	External Validation Group (%) (*n* = 85)
Age (Years)			
<40	282 (20.3)	100 (16.9)	20 (23.5)
40–59	545 (39.2)	263 (44.4)	47 (55.3)
≥60	562 (40.5)	229 (38.7)	18 (21.2)
Sex			
Female	832 (59.9)	351 (59.3)	52 (61.2)
Male	557 (40.1)	241 (40.7)	33 (38.8)
Race			
White	1178 (84.8)	514 (86.8)	0 (0)
Black	129 (9.3)	38 (6.4)	0 (0)
Asian or Pacific Islander	77 (5.5)	34 (5.7)	85 (0)
American Indian/Alaska Native	5 (0.4)	6 (1.0)	0 (0)
Tumor Size (mm)			
≤20	736 (53.0)	290 (49.0)	54 (63.5)
20–40	392 (28.2)	172 (29.1)	23 (27.1)
≥40	261 (18.8)	130 (21.9)	8 (9.4)
Stage			
I	542 (39.0)	210 (35.5)	24 (28.2)
II	291 (21.0)	135 (22.8)	11 (12.9)
III	140 (10.1)	66 (11.1)	18 (21.2)
IV	416 (29.9)	181 (30.6)	32 (37.6)
T Stage			
T1	672 (48.4)	257 (43.4)	49 (57.6)
T2	323 (23.3)	165 (27.9)	25 (29.4)
T3	284 (20.4)	122 (20.6)	5 (5.9)
T4	110 (7.9)	48 (8.1)	6 (7.1)
N Stage			
N0	857 (61.7)	359 (60.6)	35 (41.2)
N1a	166 (12.0)	77 (13.0)	18 (21.2)
N1b	366 (26.3)	156 (26.4)	32 (37.6)
M Stage			
M0	1297 (93.4)	547 (92.4)	77 (90.6)
M1	92 (6.6)	45 (7.6)	8 (9.4)

**Table 2:** Univariate and multivariate Cox analysis results for the OS of MTC patients.

Variable	Univariate Survival Analysis			Multivariate Survival Analysis		
	HR	95% CI	*p*-Value	HR	95% CI	*p*-Value
Age (Years)						
<40	Reference			Reference		
40–59	2.558	1.540–4.250	<0.001	2.920	1.750–4.872	<0.001
≥60	7.354	4.543–11.905	<0.001	7.610	4.674–12.392	<0.001
Sex						
Female	Reference			Reference		
Male	1.743	1.398–2.172	<0.001	1.011	0.797–1.283	0.928
Race						
White	Reference					
Black	0.904	0.613–1.334	0.611	-	-	-
Asian or Pacific Islander	0.405	0.201–0.818	0.012	-	-	-
American Indian/Alaska Native	2.160	0.537–8.686	0.278	-	-	-
Tumor Size						
≤20	Reference			Reference		
20–40	1.651	1.253–2.174	<0.001	2.338	1.219–4.483	0.011
≥40	3.386	2.605–4.401	<0.001	3.330	1.826–6.073	<0.001
Stage						
I	Reference					
II	1.299	0.900–1.873	0.162	-	-	-
III	1.275	0.794–2.048	0.314	-	-	-
IV	4.241	3.201–5.620	<0.001	-	-	-
T Stage						
T1	Reference					
T2	1.299	0.942–1.792	0.110	-	-	-
T3	2.508	1.889–3.328	<0.001	-	-	-
T4	5.136	3.753–7.030	<0.001	-	-	-
N Stage						
N0	Reference			Reference		
N1a	1.612	1.136–2.287	0.007	1.327	0.981–1.793	0.066
N1b	3.007	2.373–3.809	<0.001	1.874	1.493–2.351	<0.001
M Stage						
M0	Reference			Reference		
M1	8.317	6.364–10.870	<0.001	3.868	2.805–5.335	<0.001

## Data Availability

The data of the modeling group and the internal verification group were all from online sources; see the SEER database’s official website (http://seer.cancer.gov/data/, accessed on 8 August 2023). The data of the external verification group were all from the medical record information system of the First Affiliated Hospital of Zhejiang University School of Medicine. The obtained data were approved by the Ethics Committee.
